# Bioprospecting of a Novel Plant Growth-Promoting Bacterium *Bacillus altitudinis* KP-14 for Enhancing *Miscanthus* × *giganteus* Growth in Metals Contaminated Soil

**DOI:** 10.3390/biology9090305

**Published:** 2020-09-22

**Authors:** Kumar Pranaw, Valentina Pidlisnyuk, Josef Trögl, Hana Malinská

**Affiliations:** 1 Department of Environmental Chemistry and Technology, Faculty of Environment, Jan Evangelista Purkyně University in Ústí nad Labem, 40096 Ústí nad Labem, Czech Republic; Valentyna.Pidlisniuk@ujep.cz (V.P.); Josef.Trogl@ujep.cz (J.T.); 2 Department of Environmental Microbiology and Biotechnology, Institute of Microbiology, Faculty of Biology, University of Warsaw, Miecznikowa 1, 02-096 Warsaw, Poland; 3 Department of Biology, Faculty of Science, Jan Evangelista Purkyně University in Ústí nad Labem, 40096 Ústí nad Labem, Czech Republic; malinskah@gmail.com

**Keywords:** abiotic stress, lead tolerance, *Bacillus altitudinis*, P-solubilization, *Miscanthus* × *giganteus*, post-mining metal-contaminated soil

## Abstract

**Simple Summary:**

Marginal land represents poor soil with low agricultural characteristics and crop productivity, which is sometimes additionally contaminated. The exploitation of marginal land for normal agriculture is not possible but it suitable for cultivation of energy crops, especially *Miscanthus* × *giganteus* (*Mxg*), however, the harvest biomass value in such land is lower. The produced *Mxg* biomass can be converted to alternative energy like biofuel and biogas, or used for production of other value-added products like insulation fibers, building materials or paper, etc. It is well known fact that plant growth-promoting bacteria are beneficial for stimulating the overall development of plants even under stress conditions. In the current study, a number of strains were isolated from the metal-contaminated post-mining land, identified, biochemically characterized, and evaluated for abiotic stress tolerance: pH, temperature, salinity, and heavy metal (lead). Among different isolates, the multiple abiotic stress-tolerant plant growth-promoting bacteria *Bacillus altitudinis* KP-14 showed the best properties. Its effect on the growth of *Mxg* under the severe stress of metal-contaminated soil was evaluated. It was found that selected bacterial strain KP-14 significantly enhanced the biomass production. The overall results suggested that *B. altitudinis* KP-14 can be recommended as a potent biofertilizer for marginal lands.

**Abstract:**

Use of plant growth-promoting bacteria (PGPB) for cultivation of the biofuel crop *Miscanthus* × *giganteus* (*Mxg*) in post-military and post-mining sites is a promising approach for the bioremediation of soils contaminated by metals. In the present study, PGPB were isolated from contaminated soil and screened for tolerance against abiotic stresses caused by salinity, pH, temperature, and lead (Pb). Selected strains were further assessed and screened for plant growth-promoting attributes. The isolate showing the most potential, *Bacillus altitudinis* KP-14, was tested for enhancement of *Mxg* growth in contaminated soil under greenhouse conditions. It was found to be highly tolerant to diverse abiotic stresses, exhibiting tolerance to salinity (0–15%), pH (4–8), temperature (4–50 °C), and Pb (up to 1200 ppm). The association of *B. altitudinis* KP-14 with *Mxg* resulted in a significant (*p* ≤ 0.001) impact on biomass enhancement: the total shoot and dry root weights were significantly enhanced by 77.7% and 55.5%, respectively. The significant enhancement of *Mxg* biomass parameters by application of *B. altitudinis* KP-14 strongly supports the use of this strain as a biofertilizer for the improvement of plant growth in metal-contaminated soils.

## 1. Introduction

The marginal land term generally indicates land with poor soil characteristics and low crop productivity with no potential in terms of economic perspective. There are diverse factors behind formation of marginal lands, i.e., limited rainfall, extreme temperature, low quality, steep terrain, shallow depth, reduced fertility, coarse-textured, stony, heavy cracking clays, salt-affected, waterlogged, marshy lands, barren rocky soils, heavy metals etc. Mining, smelting, and associated activities are often the cause of soil pollution by different metals [1,2]. In Europe, 28% of potential agricultural land is not fit for agriculture, and in the Czech Republic alone, this estimate is 8.6% [3]. The remediation of these soils is essential for regional sustainable development. Energy crops grown in these areas can help in reclaiming land, substantially mitigating greenhouse gases without posing any risk to food security, and in providing cellulosic biomass. The burden of marginal land has somehow become an opportunity for growing energy crop like *Miscanthus* × *giganteus* (*Mxg*) to avoid competition with food crops for agriculture land and even after posing a risk to the environment and human health these sites are recognized as having potential due to their economic perspective [4].

Different technologies have been proposed for remediation of post-mining metal-contaminated soils. However, the most commonly used methods—the physical dig and dump and chemical treatments like acid leaching, electroreclamation, etc.—are costly and laborious and can negatively impact on the environment [5]. Phytoremediation is a viable eco-friendly alternative technology that uses plants to remediate and revegetate different contaminated sites and can be applied in situ [6]. A variety of techniques and applications are included in the term phytoremediation, and are mainly differentiated by the process through which plants remove, immobilize, or degrade contaminants, e.g., phyto-immobilization (plants prevent transport of dissolved contaminants in the soil), phyto-extraction or phyto-filtration (plants extract the organic or inorganic contaminants from soil and water and store them in harvestable tissue), phytovolatilization (plants remove contaminants through volatilization) or phytostabilization (inorganic contaminants such as heavy metals in the soil are immobilized so that their further transport in water or dust is minimized), and phyto-degradation (plants mineralize or assimilate contaminants in soil or water) [6].

*Mxg* is the most perspective plant within the group of the second-generation crops [7], showing a good harvest; a C_4_ photosynthetic pathway; and a good environmental profile with the potential to increase soil carbon, nutrients, biodiversity, and possibility to reduce nutrient run-off and leaching.

*Mxg* showed a multiyear stable growth at the heavy metals contaminated land [8], including Pb-contaminated military soil [9]. However, the harvest biomass value in such contaminated soils is lower [10,11,12,13]. This fact has stimulated researches toward using agricultural and microbiological practices for increasing miscanthus biomass production. Generally, there are two main approaches to be considered. The first approach is about influence to the soil by adding different amendments such as fertilizers [14], sludge [11,15], and biosolids [9], or incorporating to the system citric acid, EDTA [16], and fungi [17]. The second approach is focused on direct impact to rhizomes, which can be achieved by co-composting treatment, treatment by plant growth regulators [18], and incorporation of the microbial organisms [19].

Plants constantly interact with microorganisms in/on of all their parts. From the phytoremediation point of view, the most important interactions are those with roots [20]. Colonization of roots with arbuscular mycorrhiza fungi leads to increased stress tolerance in metal-contaminated soil [21]. Rhizosphere bacteria can enhance biodegradation of organic pollutants [22,23,24]. From the agriculture point of view, plant growth-promoting bacteria (PGPB) are beneficial for stimulating the overall development of plants.

PGPB provide an option to deal with different abiotic stresses like temperature, pH, and heavy metal contamination [25]. Application of PGPB can enhance the growth of plants under stress conditions by regulating the nutritional and hormonal balance, solubilizing nutrients (P, Zn, and K), inducing the production of growth regulators (1-aminocyclopropane-1-carboxylate (ACC) deaminase and indole acetic acid (IAA)), as well as protecting from plant pathogens by producing hydrogen cyanide (HCN) and siderophore [26]. The majority of PGPB strains belong to the genera Bacillus, Pseudomonas, Enterobacter, Burkholderia, Acinetobacter, Alcaligenes, Arthrobacter, Azospirillum, Azotobacter, Beijerinckia, Erwinia, Flavobacterium, Rhizobium, and Serratia [27]. The benefits of PGPB application include enhancing root and shoot growth, nutrient uptake, and hydration; increasing chlorophyll content; and strengthening resistance to disease [28]. The beneficial effects of PGPB have been demonstrated for many crops: wheat [29], tobacco [30], mustard [31], tomatoes and potato [32], bell peppers [33], cucumbers [34], and perennial crops such as *Miscanthus* sp. [5,35] and switchgrass [36]. PGPB have been explored as effective agents for promoting the growth of plants in contaminated sites; their positive effect is due to the enhancement of nutritional effects as well as protective effects against different stresses [5,35]. From that perspective, an association with PGPB could play an essential role in the enhancement of *Mxg* development under different stress conditions when the crop is cultivated on contaminated or marginal lands lacking nutrients.

Only a few studies have researched the impact of PGPB on the production of *Miscanthus* sp. in metal-contaminated soil [5,35]. Babu et al. applied an endophytic PGPB, *Pseudomonas koreensis*, to enhance the growth of *Miscanthus sinensis* on contaminated land; however, no statistically significant results were obtained [5]. Similarly, Schmidt et al. explored the consortium of endophytic PGPB (*Pantoea ananatis* and *Pseudomonas savastanoi*) on growth enhancement of *Mxg* under greenhouse conditions in polluted as well as non-polluted soil. In this study, application of both selected PGPB strains did not result in any significant impact on *Mxg* growth [35]. In the reported studies, the tolerance of PGPB against diverse abiotic stress factors was not evaluated. The tolerance of PGPB to temperature, pH, heavy metals, and salt is crucial for the success of application when *Mxg* is grown on severely stressed metal-contaminated soil or degraded or marginal lands (which usually have disturbed soil pH and high salinity) at field scale. In view of the above reasons, we aimed to isolate novel indigenous multiple-abiotic-stress-tolerant PGPB that could be used for enhancement of *Mxg* growth under the severe stress of metal-contaminated soil.

The present study focused on (1) isolation and characterization of novel PGPB strains tolerant to multiple abiotic stresses from metal-contaminated soil and (2) evaluation of the effect of PGPB on the biomass enhancement of *Mxg* grown in metal-contaminated soil.

## 2. Materials and Methods

### 2.1. Soil Sampling: Agrochemical and Elemental Characterization

Soil samples were collected from a metal-contaminated site in Všebořice, a suburban quarter of Ústí nad Labem, Czech Republic (50°42′11.9” N 13°58′32.1” E). The site was formerly an opencast brown coal mine operated between 1958 and 1980, which was then used as a landfill. The particular area where the soil was collected was predominantly used for dumping construction waste and was reclaimed by a thin layer of agricultural soil. Pieces of previous waste and coal can be found in the soil. Using a composite sampling approach, one square 5 m × 5 m area was selected for testing. Five samples were taken at depths of 0–30 cm (i.e., one from each corner and one from the center), mixed, and used for agrochemical and elements analyses and in the mesocosm experiment (Section 2.7). The soil samples were sieved (pore size 2 mm) to remove plant material and other particles like stones, and stored at 4 °C until use. The agrochemical characteristics were determined in accordance with standards DSTU4289-2004, 2005 and DSTU4729-2007, 2006, which are presented in Table S1. Element concentrations were measured using X-ray fluorescence analysis in accordance with the United States Environmental Protection Agency (USEPA) standard [37], with details of the analysis layout as per a previous study [8]. The concentrations of elements in the studied soil are presented in Table S2.

### 2.2. Isolation and Identification of Plant Growth-Promoting Bacteria

Collected soil (10 g) was added to 100 mL nutrient broth (5 g/L peptone and 5 g/L beef extract (pH 7.2)) supplemented with 5% NaCl and incubated at 28 °C with shaking at 120 rpm for 48 h. After incubation, the flask was maintained in a stagnant position for 1 h to allow the soil to settle. Using the spread plate technique, 100 µL of supernatant was inoculated over two different media: nutrient agar medium (5 g/L peptone, 3 g/L NaCl, 5 g/L beef extract, and 16 g/L agar (pH 7.2)) and the more nutrient-rich King’s B medium (20 g/L peptone, 1.5 g/L MgSO_4_^·^7H_2_O, 1.8 g/L K_3_PO_4_^·^3H_2_O; 10 mL/L glycerin, and 16 g/L agar (pH 7.0)). Nutrient-rich King’s B medium was used to boost the growth of microbes that require more nutrition for growth. Different morphotypes showing moderate growth were selected for further purification, and their morphological characteristics were studied using the Gram staining technique. The biochemical profiles of selected isolates were characterized using HiCarbo kit KB009 (HiMedia Laboratories, Mumbai, India), which is a comprehensive test system combining 35 tests for utilization of different carbohydrates [38].

#### 16S rDNA Sequencing and Phylogenetic Analysis

All selected isolates were subjected to taxonomic and phylogenetic analyses by 16S rDNA sequencing. Cultures were grown in 10 mL nutrient broth for 24 h, and genomic DNA was isolated using Quick-DNA Fungal/Bacterial Microprep Kit (Zymo Research, Irvine, CA, USA) following the manufacturer’s instructions. PCR assays were carried out to amplify the 16S rDNA region using the following universal bacterial 16S rDNA primers; forward (8F-AGAGTTTGATCCTGGCTCAG) and reverse (1542R-AAGGAGGTGATCCAGCCGC A) [39]. PCR amplification was carried out in a 25 µL reaction mixture containing 1 µL genomic DNA (30–50 ng), 1.5 µL of 20 µM forward and reverse primers in a 1:1 mixture, 12.5 µL PCR master mix (MyTaq^TM^ Mix, Bioline, London, UK), and 10 µL nuclease-free water. The PCR reaction was performed using the following amplification conditions; initial denaturation of 3 min at 95 °C; followed by 34 cycles for 60 s at 95 °C, 30 s at 61 °C, and 1 min 30 s at 72 °C; and a final extension period of 5 min at 72 °C. The amplified PCR product was resolved by horizontal electrophoresis in 1.2% agarose gel in 1× TAE (Tris-acetate-EDTA) buffer and purified using the PCR purification kit (SureCleanPlus, Bioline, London, U.K.) following the manufacturer’s protocol. The purified PCR products were further sequenced using the 16S rDNA bacterial universal primers and the Applied Biosystems ABI prism 310 automated DNA sequencer (Foster City, CA, USA) at the DNA Sequencing Center, Institute of Microbiology of the Academy of Sciences of the Czech Republic, Prague. The 16S rDNA sequences were approximately 1.5 kb in length.

The partial 16S rDNA sequences of the isolates were compared with sequences available from the National Center for Biotechnology Information (NCBI; USA; http://www.nvbi.nlm.nih.gov) nucleotide nr/nt (non-redundant nucleotide) database to identify the nearest taxa. Multiple sequence alignments were performed using ClustalW online software. To calculate evolutionary distances, phylogenetic dendrograms were constructed by the neighbor-joining method based on 16S rDNA sequences, and tree topologies were evaluated by 1000 datasets using online software ETE3 (https://www.genome.jp/tools-bin/ete) [40].

### 2.3. Screening of Isolated Bacterial Strains for Abiotic Stress Tolerance Ability

All bacterial isolates were screened for different abiotic stress tolerances, including salt, temperature, and heavy metal, using the respective medium. After incubation, the growth of isolates under different abiotic stress conditions was compared with those grown on standard nutrient agar plates at 28 °C. After comparison with the control plates, results were classified as negative (−) for no growth, moderately positive (++) when there was more growth though still less than the control plates, and strongly positive (+++) for growth similar to the control plates. All experiments were carried out in triplicates and each assay repeated at least two times.

#### 2.3.1. Salt Tolerance

For determining the salt tolerance of different isolates, nutrient agar plates with increasing concentrations of NaCl from 0% to 20% were prepared and inoculated with different isolates and incubated at 28 °C for 48–96 h [39].

#### 2.3.2. Temperature Tolerance

For examining temperature tolerance, isolates were streaked on nutrient agar medium and incubated at different temperatures of 4, 10, 16, 22, 28, 37, 40, 45, and 50 °C for 48–96 h [41].

#### 2.3.3. pH Tolerance

For determining the pH tolerance profile of different isolates, nutrient agar media with different pH ranges between 4 and 8 were prepared. The final pH of the medium was adjusted using 1 M HCl or 1 M NaOH. The agar concentration was increased to 20 g/L at pH 4. All isolates were streaked over the nutrient agar medium and kept for incubation at 28 °C for 48–96 h.

#### 2.3.4. Heavy Metal Tolerance

All isolates were inoculated on nutrient agar medium spiked with different concentrations of Pb(NO_3_)_2_: 100, 300, 500, 700, 1000, 1200, and 1500 ppm. After inoculation, plates were incubated at 28 °C for 48–96 h [42].

### 2.4. In Vitro Assessment of Plant Growth-Promoting (PGP) Attributes

Based on their abiotic stress tolerance, the three isolates—KP-14, KP-18, and KP-19—were selected for further assessment of their PGP attributes. The phosphate solubilization potential of the selected isolates was evaluated both qualitatively and quantitatively. Another important PGP trait, indole acetic acid (IAA) production, was also evaluated by quantitative estimation. The remaining PGP traits, i.e., siderophore production, hydrogen cyanide (HCN) production, and ammonia production, were only evaluated qualitatively. All experiments were carried out in triplicates and each assay repeated at least two times.

#### 2.4.1. Inorganic Phosphate Solubilization

The phosphate solubilization potential of all isolates was qualitatively estimated using NBRIP medium (National Botanical Research Institute’s phosphate growth medium) (10 g/L glucose, 5 g/L MgCl_2_·6H_2_O, 0.25 g/L MgSO_4_·7H_2_O, 0.2 g/L KCl, 0.1 g/L (NH_4_)_2_·SO_4_, 5 g/L Ca_3_(PO_4_)_2_, 16 g/L agar (pH 7.2)) [43]. NBRIP plates were spot-inoculated with 3 µL cultures grown for 24 h (optical density (OD) ~ 0.6) and incubated at 28 °C for 4–5 days. The presence of a halo zone around the areas of bacterial growth indicates the P solubilization potential of the particular strain [44]. P solubilization of the isolates was quantitatively estimated using the NBRIP broth medium. In a 100 mL Erlenmeyer flask, 40 mL of NBRIP broth medium was sterilized and inoculated with 2% inoculum of freshly grown isolates (OD~0.6). Inoculated flasks were incubated at 28 °C and agitated at 120 rpm for 10 days. We withdrew a 2 mL sample at regular 2 day intervals to spectrophotometrically monitor the concentration of soluble phosphorus using the molybdenum blue method [43,44,45]. The amount of solubilized P was obtained by subtracting the soluble P of the inoculated sample from the corresponding uninoculated control sample (i.e., P released during autoclaving of the medium).

#### 2.4.2. IAA Production

The production of indole acetic acid (IAA) was estimated using 50 mL Jensen’s broth (20 g/L sucrose, 1 g/L K_2_HPO_4_, 0.5 g/L MgSO_4_·7H_2_O, 0.5 g/L NaCl, 0.1 g/L FeSO_4_·7H_2_O, 0.005 g/L Na_2_MoO_4_·2H_2_O, 2 g/L CaCO_3_ (pH 7.2)) containing 5 mM tryptophan inoculated with 2% (v/v) of inoculum and kept for incubation at 28 °C at 120 rpm for 7 days. Cultures were centrifuged at 10,000× *g* for 10 min, and supernatants were used for the quantification of IAA. Salkowski reagent (0.5 M FeCl_3_ + 60% sulfuric acid) was added to the culture supernatant at a 4:1 ratio, and the absorbance (OD) was measured at 530 nm. The concentration of IAA was calculated using the standard curve of pure indole-3-acetic acid (Sigma Aldrich, St. Louis, MO, USA) [29,46]. The amount of IAA produced was obtained by subtracting the IAA of the inoculated sample from the corresponding uninoculated control sample.

#### 2.4.3. Siderophore Production

Siderophore production was estimated using the modified chromazurol S (CAS) (Sigma Aldrich, St. Louis, MO, USA) agar assay developed by Schwyn and Neilands [47]. CAS dye solution is a mixture of three different solutions: Solution I containing 60 mg chromazurol S dye in 50 mL distilled water, Solution II containing 1 mM FeCl_3_·6H_2_O in 10 mM HCl, and Solution III containing 72.9 mg of CTAB (Cetyl trimethylammonium bromide) in 40 mL distilled water. All three solutions were mixed to prepare the 100 mL CAS dye solution, which were then sterilized at 121 °C for 15 min. For preparation of chromazurol S agar plates, 100 mL sterilized CAS dye solution was mixed with 300 mL sterilized nutrient broth immediately before pouring into Petri plates. All cultures were inoculated over CAS agar medium and incubated at 28 °C for 48 h. The occurrence of a yellow halo around the bacterial colony is a positive indicator of siderophore production [44].

#### 2.4.4. Hydrogen Cyanide (HCN) Production

HCN production by the isolates was qualitatively estimated following the Bakker and Schipper method [48]. The bacterial isolates were streaked over the King’s B agar plate containing 4.4 g/L glycine. A single sheet of Whatman filter paper No. 1 (90 mm) was soaked in 0.5% picric acid in 2% sodium carbonate and the soaked disc was placed in the lid of each Petri plate. All Petri plates were sealed with parafilm and incubated at 28 °C for 4 days. An uninoculated control was included for comparison of results. After incubation, positive results were confirmed by a color change of the filter paper from deep yellow to orange and finally to orange-brown to dark brown. A negative test is indicated by the deep yellow color of the filter paper remaining unchanged after the growth of bacteria.

#### 2.4.5. ACC (1-Aminocyclopropane-1-Carboxylate) Deaminase Activity

Isolates with ACC deaminase activity are able to assimilate ACC for their growth. Based on this, ACC deaminase activity was qualitatively examined for all three isolates using modified nitrogen-free DF (Dworkin and Foster) salt medium (10 g/L glucose, 0.41 g/L KH_2_PO_4_, 0.52 g/L K_2_HPO_4_, 0.05 g/L Na_2_SO_4_, 0.2 g/L CaCl_2_, 0.1 g/L MgSO_4_·7H_2_O, 0.005 g/L FeSO_4_·7H_2_O, 0.002 g/L Na_2_MoO_4_·2H_2_O (pH 7.1)) amended with 3 mM ACC as the sole nitrogen source. (NH_4_)_2_·SO_4_ (0.2% *w*/*v*) was added as the sole nitrogen source in the control plate [49]. Both control and ACC-supplemented plates were spot-inoculated with all three selected isolates and incubated at 28 °C for 3–5 days. ACC deaminase activity of isolates was confirmed by their growth on ACC-supplemented plates. Negative results were confirmed by their growth on control plates [50].

#### 2.4.6. Ammonia Production

For qualitative estimation of ammonia production, bacterial isolates were grown in 10 mL peptone water (10 g/L peptone and 5 g/L NaCl (pH 7.0)) in test tubes and incubated at 28 °C at 120 rpm for 4 days. Following incubation, 1 mL Nessler’s reagent was added to each tube. Due to reaction between ammonia and Nessler’s reagent, peptone water color changed from faint yellow to brownish based on the ammonia concentration. The presence of a faint yellow color indicates a small amount of ammonia, and deep yellow to brownish colors indicate higher ammonia production [51]. A negative control with uninoculated peptone water was also used to compare the results.

### 2.5. In Vitro Antagonistic Bioassays against Plant Pathogenic Fungi

Two plant pathogenic fungi—*Fusarium culmorum* (CCF-1745) and *Botrytis cinerea* (CCF-2361)—were obtained from the Culture Collection of Fungi (CCF), Department of Botany, Faculty of Science, Charles University, Prague, Czech Republic. The antifungal activity of the selected bacteria was assessed against both plant pathogenic fungi on potato dextrose agar (PDA) medium (200 g/L infusion from potatoes and 20 g/L glucose (pH 5.7)) [44]. For routine culture, fungi were grown on PDA medium for 7 days at 25 °C. After 7 days of incubation, 1 cm plugs of mycelia of actively growing fungus were equidistantly placed at each side of the streak of a different bacterial strain and incubated for 7 days at 25 °C. After incubation, antagonistic activity was observed on the basis of the mycelial growth inhibition from different isolates. The experiment was performed in triplicate for each combination of fungus and bacterial strain.

### 2.6. Seed Germination Assay

Based on the in vitro plant growth-promoting assays, the bacterial isolate with the most potential, KP-14, was selected and tested for its efficacy in augmenting seed germination. The selected bacterial isolate was bioassayed for its ability to promote/inhibit seedling growth following the method of Shende et al. with minor modifications, i.e., 0.1% HgCl_2_ was used here as surface sterilizing agent [52]. *Brassica alba* seeds were selected for the seed germination assay and collected from the local market. Seeds were surface sterilized with 0.1% HgCI_2_ for 3 min, followed by successive washings (at least 10) with sterile distilled water to remove traces of HgCl_2_. For bacterization, sterilized seeds were soaked in culture grown for 24 h in nutrient broth (OD~0.6) for ~1 h. For the control, sterilized water was used instead of broth culture. Thereafter, 10 seeds per replicate were placed on soft agar plates (0.8% agar) using sterile forceps and incubated at 25 °C in the dark. After 3 days, the percentage of seed germination (% seed germination or germination index (GI) = (seeds germinated/total no. of seeds) × 100) and lengths of the root and shoot were measured. Five replicates of each treatment were used in this experiment.

### 2.7. Mxg Growth in Metal-Contaminated Soil

The effect of selected isolate KP-14 on *Mxg* growth was evaluated with the mesocosm experiment using pots (30 cm top and 20 cm bottom diameters, 25 cm in height) containing 2 kg of metal-contaminated soil. A completely randomized block design with four replications was used. The rhizomes of *Mxg* were received from the deposit field in Zagreb, Croatia and were three years old. Rhizomes were cleaned with distilled water to remove residual soil and cut into pieces. A single piece with an average size of 10 cm containing two buds was used for bacterization before planting in each pot. For bacterization, rhizomes were dipped in a bacterial suspension (10^7^ cells/mL) of isolated strain KP-14 for 1 h. For preparation of the bacterial suspension, bacterial culture was grown in nutrient broth medium for 24 h at 28 °C and cells were harvested by centrifugation at 6000 rpm for 10 min. Approximately 10^8^ cells/mL were resuspended in sterilized distilled water along with 1% carboxymethyl cellulose (to increase the stickiness of the bacterial suspension). For the control pots, distilled water was used instead of the bacterial suspension along with 1% carboxymethyl cellulose. Pots with planted rhizomes were placed in the greenhouse under natural light at 28–30 °C. The soil in the pots was irrigated with tap water to maintain soil moisture. Plant growth was monitored by measuring height at regular intervals during the entire vegetation period of 6 months. After that period, plants were harvested: different plant parts (shoots, leaves, and roots) were collected separately. Plant parts were cleaned with distilled water and dried until a constant weight at 28–30 °C and shoot, leaves, and root weights were then measured.

### 2.8. Statistical Analysis

All data related to in vitro seed germination (with five replicates) and in vivo mesocosm experiment (with four replicates) were subjected to one-way standard analysis of variance (ANOVA) using Microsoft Excel 2013 (Albuquerque, NM, USA). The differences were considered significant at *p* ≤ 0.05. The biochemical experiments were carried out in triplicate, and the results are expressed as mean ± standard error (SE) values.

## 3. Results

### 3.1. Soil Agrochemical and Elemental Analyses

The pH of the collected soil indicated it was acidic in nature when measured by water and salt extraction methods: 5.0 and 3.7, respectively. The soil electric conductivity and general salt content were 0.1 mS/cm and 48 mg/100 g, respectively. Inorganic nitrogen in the form of NO_3_ and NH_4_ was found to be approximately 21.4 and 108 mg/kg, respectively; the organic matter in the collected soil was 3.4%. The detailed soil agrochemical characteristics are provided in Table S1. Analysis of the different elements in soil was also conducted. V, Cr, Ni, Zn, and Sr were detected at very high concentrations (Table S2).

### 3.2. Identification and Biochemical Characterization of Different Isolates

All isolates were identified on the basis of their partial 16S rDNA sequences. Only sequences with a similarity value of ≥98.0% were considered for identification. The results showed that isolates mainly belonged to different species of the Bacillus, Pseudomonas, Achromobacter, and Stenotrophomonas genera (Table 1).

**Figure 1:**
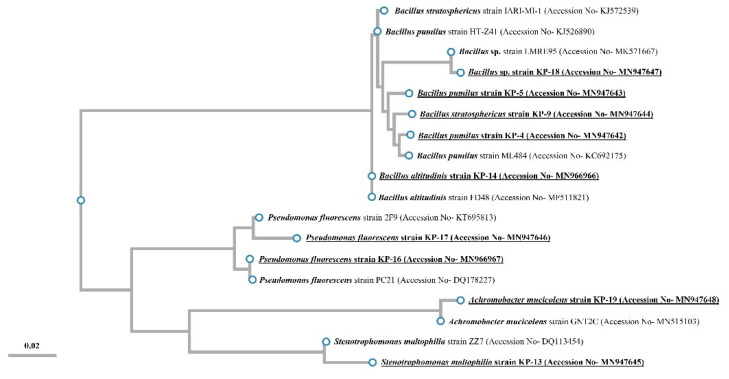
Phylogenetic trees based on 16S rRNA gene sequences showing clustering of all 9 isolates with their nearest phylogenetic relatives. Phylogenetic trees were constructed by the neighbor-joining method. The bar represents 0.02 substitutions per alignment position.

The nucleotide sequences were submitted to the NCBI GenBank database, and an accession number assigned for each sequence. These sequences were further aligned by multiple sequence alignment, and a phylogenetic tree with representative strains of related taxa was constructed using an online tool (phylogenetic analysis pipeline by ETE3) as shown in Figure 1. In Figure 1, it can be seen that all (*n* = 9) sequences clustered with their nearest neighborhood sequence as obtained via NCBI BLAST (Basic Local Alignment Search Tool) searching. The obtained sequences clustered into three taxonomic divisions including Firmicutes (Bacillus genus), gamma-proteobacteria (Stenotrophomonas genus), and beta-proteobacteria (Pseudomonas and Achromobacter genera). These results show that the isolates belong to a diverse range of bacterial divisions.

Carbon source utilization was determined to investigate the ability of bacteria to ferment and use different carbon sources. Out of 35 different carbon sources, only five carbon sources (L-arabinose, mannose, esculin hydrolysis, citrate, and malonate) were used by most of the isolated strains (Table 2).

### 3.3. Abiotic Stress Tolerance of Different Isolates

All isolates were grown over a broad temperature range of 4 to 50 °C. Most of the isolates were able to grow in the mesophilic range (20–45 °C). Among all the isolates, only five (KP-13, KP-14, KP-16, KP-17, and KP-18) showed psychrophilic behavior (i.e., were able to grow at 4–16 °C). In the moderate thermophilic range, i.e., 50 °C, only three isolates (KP-14, KP-18, and KP-19) were able to grow (Table 3). The tolerance to salinity in terms of NaCl percentage was examined. We found that all isolated strains were able to withstand NaCl concentrations up to 10%, confirming their halotolerant nature. In addition, out of nine different bacterial isolates, only *Bacillus* sp. strain KP-14 and *Achromobacter* sp. strain 19 were true halophiles by nature, able to grow at NaCl concentrations up to 15% (Table 3). All bacterial isolates were capable of growing under a broad range of pH from an acidic medium at pH 5 to alkaline at pH 8. We observed that strains KP-4, KP-5, KP-9, and KP-18 could withstand and grow under highly acidic conditions at pH 4. The tolerance of isolates to metal toxicity was screened using Pb(NO_3_)_2_. We observed that all isolates were tolerant up to 700 ppm of Pb except KP-19. KP-18 and KP-14 were able to grow at a higher (1200 ppm) concentration of Pb (Table 3).

Summarizing the abiotic stress tolerance of all researched strains, we concluded that three of the isolated strains, KP-14, KP-18, and KP-19, demonstrated an extensive abiotic stress tolerance range: temperature (4 to 50 °C), salinity (0–15% NaCl), pH (5–8), and Pb (100–1200 ppm). Based on this evaluation, the three strains KP-14, KP-18, and KP-19 were selected for further investigation.

### 3.4. Plant Growth-Promoting Characteristics of the Selected Isolates

Table 4 lists the various plant growth-promoting activities of the selected isolates KP-14, KP-18, and KP-19. The investigation illustrated that the three strains produced positive results for phosphate solubilization, IAA, ammonia, and siderophore production (Table 4).

#### 3.4.1. Phosphate Solubilization

**Figure 2:**
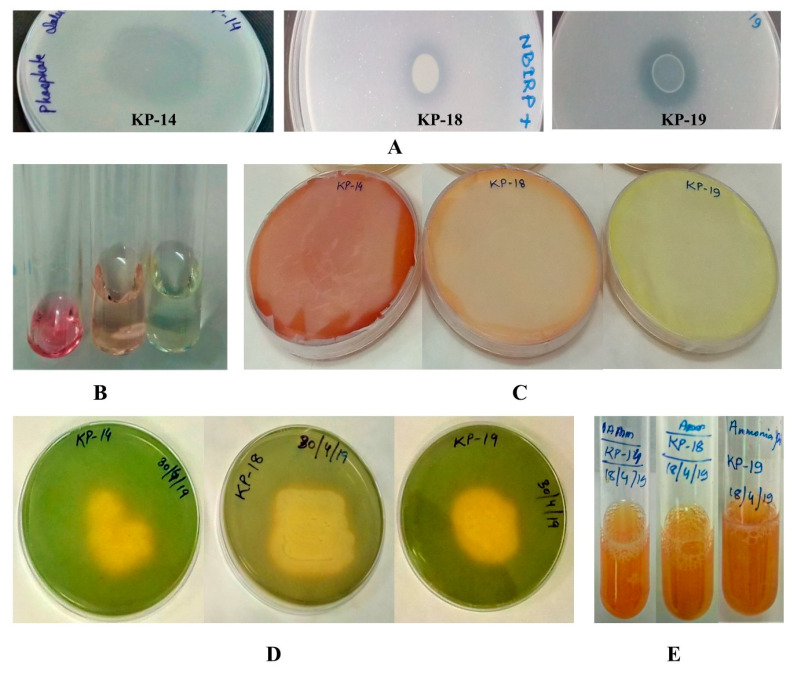
Evidence of different plant growth promoting (PGP) attributes for selected isolates KP14, KP18, and KP19: (**A**) phosphate solubilization, confirmed by the clear halo around the culture; (**B**) IAA production, confirmed by the pink color development in the reaction mixture; (**C**) HCN production (brown color development in the picric acid-soaked filter paper); (**D**) siderophore production, confirmed by yellow halo on CAS agar plates; and (**E**) ammonia production, confirmed by the brown color development in the reaction mixture.

The selected isolates (KP-14, KP-18, and KP-19) were first qualitatively examined for their potential to convert the inorganic form of phosphorus into the soluble form. A clear halo around the colony was observed on 2% TCP (Tri calcium phosphate)-supplemented NBRIP agar medium (Figure 2A), which was further supported by the quantitative estimation of solubilized P in NBRIP liquid medium. P solubilization was observed in all three selected strains: KP-14 at 130 µg/mL, KP-18 at 14.5 µg/mL, and KP-19 at 32.8 µg/mL. In the case of P-solubilization, KP-14 showed the most potential with maximum activity in both qualitative (3.7 mm halo zone) and quantitative (130 ± 3.2 µg/mL of soluble phosphate) analyses after 4 days of incubation (Table 4).

#### 3.4.2. IAA Production

Biosynthesis of IAA from tryptophan-supplemented media showed a major difference among the selected strains. The development of a pink color confirmed the presence of IAA in the supernatant (Figure 2B); for quantification, the intensity of the pink color was measured spectrophotometrically at 530 nm. The quantitative production of IAA ranged between 0.5 and 27.8 µg/mL; the IAA production was highest in strain KP-14 after 6 days of incubation (Table 4).

#### 3.4.3. HCN Production

The HCN production was strongly positive for isolate KP-14 and slightly positive for KP-18 strain, whereas the yellow color of picrate-soaked filter paper did not turn brick red-brown for isolate KP-19 (Figure 2C).

#### 3.4.4. Siderophore Production

In terms of siderophore production, the isolates displayed a change in shading from greenish-blue CAS agar media to yellow, indicating significant generation of siderophore (Figure 2D).

#### 3.4.5. Ammonia and ACC Deaminase Production

Ammonia (NH_3_) production was evaluated for selected isolates. We found that all three isolates produced ammonia, confirmed by the brown color development in the reaction medium (Figure 2E). ACC deaminase production was observed in only one strain, KP-14. KP-14 was able to grow on DF minimal medium supplemented with 3 mM ACC as a sole nitrogen source, implying positive ACC deaminase activity.

#### 3.4.6. In Vitro Antagonistic Bioassays against Plant Pathogenic Fungi

**Figure 3:**
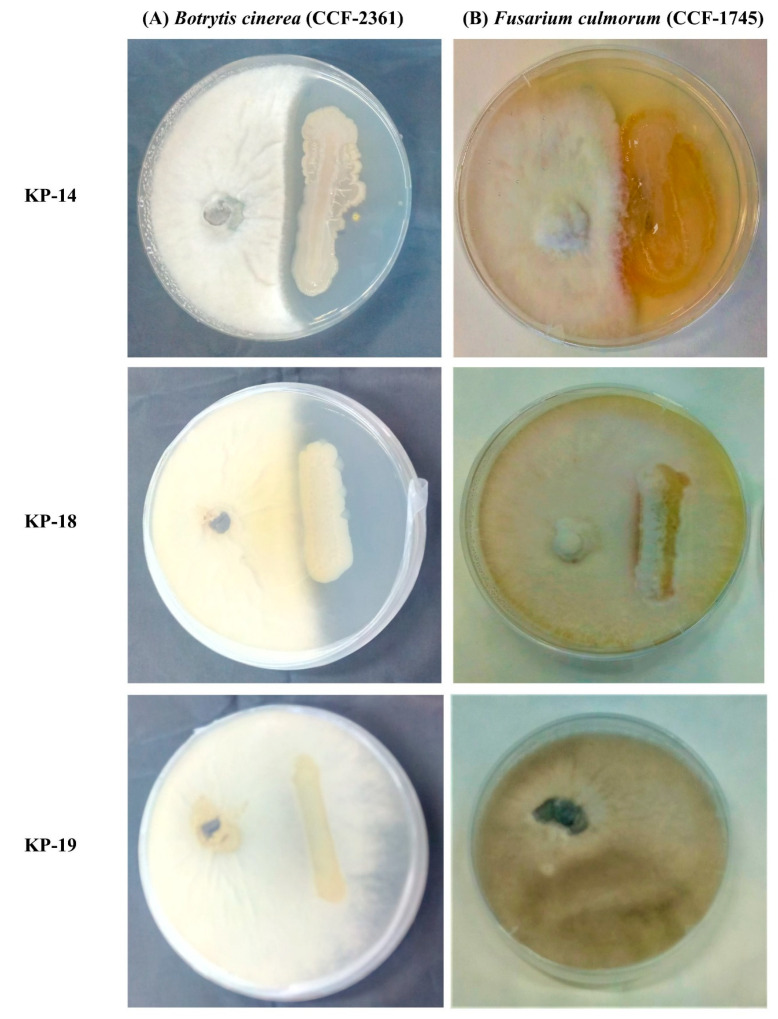
Antagonistic activity of selected isolates KP-14, KP-18, and KP-19 against (**A**) *Botrytis cinerea* (CCF-2361) and (**B**) *Fusarium culmorum* (CCF-1745).

The antagonistic activities of the three selected isolates against two plant pathogenic fungi *(Fusarium culmorum* (CCF-1745), and *Botrytis cinerea* (CCF-2361)) were investigated using the in vitro dual culture growth inhibition method. After 7days of incubation, it was observed that out of three selected isolates, only *Bacillus altitudinis* KP-14 successfully inhibited the mycelial growth of both *F. culmorum* and *B. cinerea* at a certain level (Figure 3), whereas *Bacillus* sp. KP-18 exhibited inhibition only against *F. culmorum*. In addition, *Achromobacter* sp. KP-19 was found to be susceptible against both tested plant pathogens.

#### 3.4.7. Effect of *B. altitudinis* KP-14 on Germination of *Brassica alba* Seeds

**Figure 4:**
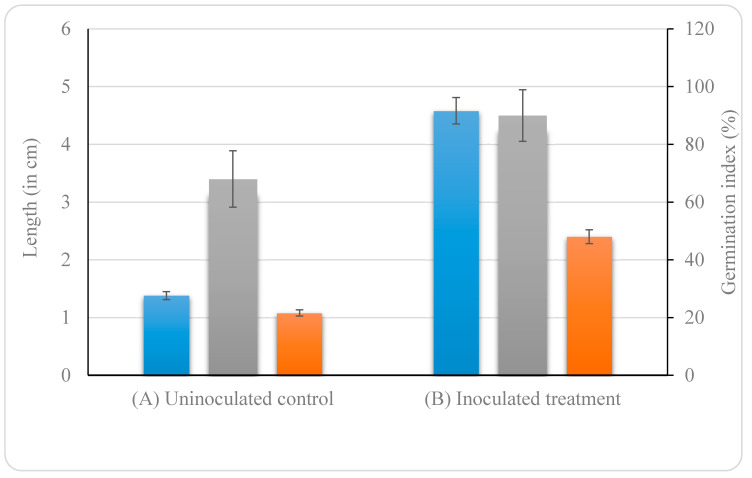
Effect of *B. altitudinis* KP-14 on germination of *Brassica alba* seeds: (**A**) uninoculated control and (**B**) inoculated treatment. Displayed data is the mean ± SE value of five replicates of each treatment. (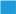 root length, 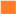 shoot length, and 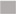 germination index (%)).

Based on the above observations, *B. altitudinis* KP-14 was selected for the seed germination study. The results show that the germination of *Brassica alba* seeds was significantly enhanced (*p* < 0.02) by the inoculation of the bacterial isolate (Table S3). In the control (uninoculated treatment), the germination index was ~68%, whereas in the bacteria-inoculated treatment, a 32.5% increase was observed, which corresponds to a germination index of ~90%. The root and shoot lengths were examined after 3 days of growth. Similarly, the bacterization of seeds significantly affected (*p* < 0.001) root growth and shoot length (Figure 4) (Table S4). The highest root and shoot lengths (6.2 and 3.0 cm, respectively) were observed in PGPB-treated seeds.

### 3.5. In Vivo Mxg Growth Promotion by B. altitudinis KP-14

**Figure 5:**
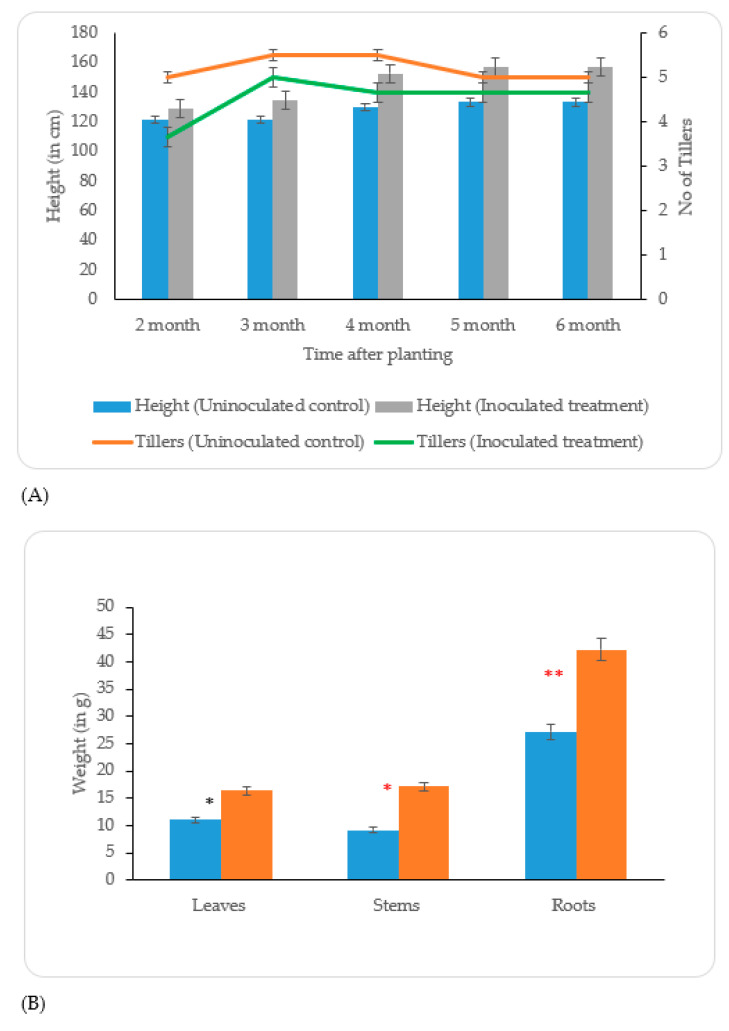
Effect of *B. altitudinis* KP-14 on *Mxg* plant growth parameters of (**A**) height and number of tillers during the vegetation period and (**B**) dry biomass of leaves, stems, and roots at harvest (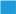 Uninoculated control; 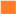 Inoculated treatment). Displayed data is the mean ± SE value of four replicates of each treatment. Asterisks denote significant differences between control and strain KP-14 experiment with four replicates (ANOVA, * *p* < 0.001, ** *p* < 0.0001).

The results of the mesocosm experiment with the growth of *Mxg* in metal-contaminated soil showed a significant effect of the PGPB inoculant *B. altitudinis* KP-14 on the following plant growth parameters: (1) plant height and (2) stems, leaves, and roots dry weights (Figure 5A). The plants treated with PGPB were taller (*p* < 0.001) than uninoculated control plants. For PGPB-treated plants, the maximum height, 168 cm, was observed after the fourth month of the growing period; thereafter, the height of the plants remained constant until harvest. Although the number of tillers was slightly less in PGPB-treated plants in comparison with the non-treated plants, this difference was statistically insignificant (*p* = 0.54) (Table S5). PGPB treatment significantly enhanced (*p* < 0.001) the roots, stems, and leaves dry weights (Figure 5B). The total shoot and dry root weights were enhanced by 77.7% and 55.5%, respectively, with respect to the uninoculated control.

## 4. Discussion

The isolation, screening, and application of multi-stress-tolerant PGPB for the improvement of plant productivity is important for meeting the nutritional requirements of plants grown in contaminated or marginal land. Therefore, we attempted to isolate some novel PGPB with tolerance against multiple abiotic stresses such as resulting from salinity, temperature, and Pb and to characterize their multiple plant-growth promoting (PGP) capabilities in terms of IAA production, phosphate solubilization, ACC deaminase production, siderophore production, ammonia production, HCN production, and antifungal activity against plant pathogens. In this study, nine different multiple-stress-tolerant bacteria were isolated and identified on the basis of their morphological, biochemical, and molecular, i.e., 16S rDNA, sequences analysis. The most stress-tolerant bacteria were further screened for their multiple PGP traits. The multiple-stress-tolerant *B. altitudinis* isolate KP-14 was screened out as having all PGP traits mentioned above, and was used in the mesocosm experiment for enhancement of *Mxg* growth under contaminated soil.

Soil pH plays a significant role in metal mobilization by controlling adsorption and precipitation processes. The pH of the collected soil was acid in nature, which was also confirmed by its low electric conductivity and general salt content [53]. Soil acidity affects plant growth in many ways, such as through the deficiency of various elements such as P, Mo, and Ca, or through the toxicity of Al, Mn, or other cations due to increasing their solubility [54]. Inorganic nitrogen in the form of NO_3_ and NH_4_ was found at 21.4 and 108 mg/kg, respectively, which is low in comparison with healthy soil [55]. Soil organic matter was 3.4%, which is in the range of regular soil (3–6%) [54,56]. The total concentrations of different elements in the studied soil are presented in Table S1. The results show that some element concentrations were higher than permissible limits. The concentrations of Zn, Ni, Cu, and Cr were 22–200% higher than their permissible limits [57]. Overall, the soil agrochemical properties and its elemental profile confirmed that normal agricultural practices cannot be used for this land and remediation is required.

Pb is one of the main toxic elements found in mining tailings, military sites, and industrial zones [12]. This element is persistent and can enter the food chain through the adsorption and accumulation in plants, disrupting the balance of the ecosystem, impairing ecological functions, and threating human health [13]. Although the Pb concentration in the collected soil was not high, the aim of the study was to develop a unique bacterial formulation that can be used for a variety of contaminated or stressed soil. This is why Pb was selected as the main research element in the current study.

Second-generation crops (*Miscanthus* sp., *Switchgrass*, etc.) have been applied in phytotechnology of the slightly-metal-contaminated or marginal soil [35,36]. The main advantages of this approach include restoration of the soil and simultaneous production of biomass. *Miscanthus* biomass contains approximately 22% lignin, 36% *α*-cellulose, and 24% hemicellulose, and can be processed into biofuel [58,59], building material [60], fiber [61], and other bioproducts [62]. However, the harvest biomass value in contaminated soils is lower [11], which has stimulated research on using agricultural and microbiological practices for increasing biomass production.

Different PGPB were isolated from post-mining metal-contaminated soil and screened for abiotic stress tolerance and PGP attributes, and were further evaluated for the enhancement of *Mxg* biomass grown in the same metal-contaminated soil. As reported, different PGP attributes of bacteria assist plants in overcoming different stresses, including P-solubilization; IAA, HCN, ammonia, and siderophore production; ACC deaminase activity; and antagonistic activity against pathogenic fungi [5,25,35].

Phosphate solubilization is a decisive characteristic of PGPB. The observations of the present study agree with numerous research findings where different microbial genera (such as Bacillus, Pseudomonas, and Halomona*s*) were reported for their solubilization of phosphate [50,63,64,65]. Besides providing phosphorous, PGPB boost plant development by increasing nitrogen fixation activity, improving the accessibility of different micronutrients, and inducing the production of plant development hormones [66]. During P solubilization by the selected isolates, there is a decrease in medium pH due to the production of organic acids such as gluconic acid, oxalic acid, and citric acid, which are responsible for P solubilization [66,67,68,69].

PGPB produce growth-regulating hormones, such as IAA, which assist the plant in the formation of roots and root hairs in addition to root elongation. The better development of the root system, in turn, promotes the water and nutrient uptake efficiency of the plants. Endogenous plant IAA, along with the PGPB-synthesized IAA, augments the secretion of plant root exudates, which serve as an energy source for the establishment of inoculated strains to improve their growth and colonization efficiency. Several strains of genus Bacillus, Azotobacter, and Pseudomonas were reported to produce IAA [44,63,70,71]. In the current research, among three selected bacterial isolates, *B. altitudinis* KP-14 showed the highest IAA production. Another important PGP attribute is ACC deaminase enzyme activity, which is one of the critical factors that facilitate the plant’s growth under stressed conditions like salinity, pH, temperature etc. In such stressed environments, plant roots secrete ACC, which is also responsible for ethylene production. The production of ethylene drastically impacts plant physiology, growth, and development. The ACC deaminase enzyme, in turn, is responsible for the dissociation of ACC into ammonia and α-ketobutyrate, and protects the plant from the negative influence of ethylene [72,73]. Therefore, ACC deaminase activity is one of the key enzymes for overcoming plant growth-related issues under different stress conditions. PGPB are known producers of ACC deaminase enzyme and we observed that out of three selected strains, only KP-14 displayed ACC deaminase activity, which is in accordance with other reports of *Bacillus* sp. (Table 5) [29,67,74].

The primary goal of this study was to isolate bacterial isolates tolerant to multiple abiotic stresses with various PGP attributes, especially novel species. *B. altitudinis* KP-14 was isolated from metal-contaminated soil and showed different PGP attributes with tolerance against multiple abiotic stresses. The isolated strain was identified on the basis of its 16S rDNA sequence and morphological and biochemical characteristics [75]. The disaccharide trehalose is a well-known stress molecule that provides protection against injuries and is produced in response to high salinity, high/low temperature, and even desiccation. Trehalose assimilation plays a significant role in the microbial colonization of plant roots. Genes involved in trehalose assimilation induce the growth of bacteria around roots and in root curls [76]. The abiotic stress tolerance behavior of the selected isolates is supported by these findings.

Another important finding of the current study was that selected isolates showed indirect PGP traits, i.e., enhancement of iron and nitrogen uptake as well as protection from plant pathogens by production of siderophore, ammonia, and HCN. All selected isolates were found to produce siderophore and ammonia. A siderophore is a low-molecular-weight iron chelator that usually binds Fe^3+^ ions and enhances the iron uptake of plants by converting the unavailable form of this element to the available form in the soil [77]. The ammonia excreted by diazotrophic bacteria is among the important characteristics of PGPB, which benefits the crop, the accumulation of ammonia in soil, supplies nitrogen to their host plant, and promotes root and shoot elongation along with increases in biomass. Ammonia and HCN suppress the growth of certain fungi and nitrobacteria as they possess a potential inhibitory effect, disturb the equilibrium of the microbial community, and inhibit the germination of spores of numerous fungi [27,31,78]. All selected isolates were positive for HCN production except for the KP-19 isolate, in agreement with the results for antifungal activity.

Antifungal activity against different plant pathogens is also an important function of PGPB and has been well documented in several studies [29,63,65]. In our study, *B. altitudinis* KP-14 and *Bacillus* sp. KP-18 showed antagonistic activity against *F. culmorum.* As shown in Figure 4, the growth of another plant pathogenic fungus, *B. cinerea*, was remarkably suppressed only by the *B. altitudinis* KP-14 strain, which was in line with the findings of previously published studies [78,79,80] reporting that *Bacillus* sp. is a potential biocontrol agent against *F. culmorum*, *B. cinerea*, and other plant pathogenic fungi. The abiotic stress tolerance and plant growth-promotion activity of different *Bacillus* sp. and data from the current study are summarized and compared in Table 5. The different types of abiotic stress tolerances with plant growth-promoting attributes show that the newly isolated *B. altitudinis* KP-14 strain is a unique and promising candidate for promotion of plant growth in metal-contaminated soils.

The positive impact of bacterial isolate *B. altitudinis* KP-14 on *Mxg* growth and biomass parameters is illustrated in Figure 5. The bacterization of *Mxg* rhizomes by *B. altitudinis* KP-14 prior to planting increased plant biomass at harvest by 77%. This improvement in biomass is due to the different PGP traits including the ACC deaminase activity, P solubilization, IAA production, siderophore production, and ammonia production capacities of *B. altitudinis* KP-14. Similar results were reported by Fei et al., who observed a significant increase in the dry shoot weight when *Miscanthus amuri* was treated with bacterial species *Gluconacetobacter diazotrophicus* PAL5T and *Azospirillum brasilense* N8 [85]. Another similar observation in a mesocosm experiment was recorded when *Mxg* was treated, before planting, with RhizoPlus^®^, which is a commercial preparation of the *Bacillus amyloliquefaciens* FZB24 strain [35]. The positive impact of PGPB on the growth of *Miscanthus sinensis* in the metal-contaminated mining soil using *Pseudomonas koreensis* AGB-1 strain was evaluated, increasing the plant biomass by 41.6% [5]. In the current study, the biomass was increased by 77.7%, which is higher than reported by Babu et al. and can be explained by the more substantial impact of the PGPB strain *B. altitudinis* KP-14 on *Mxg* development [5]. However, the difference may have partly been due to *M. sinensis* being a less productive species in comparison with *Mxg.*

## 5. Conclusions

In the present study, several PGP bacterial strains were isolated from post-mining, metal-contaminated soil and identified and characterized. Among them, the KP-14 strain, identified as *B. altitudinis,* showed stress-tolerant behavior toward multiple abiotic stresses along with several plant growth-promoting attributes such as P solubilization; production of IAA, siderophore, ammonia, and HCN; ACC deaminase activity; and antagonistic activity against two agriculturally important plant pathogens: *Fusarium culmorum* (CCF-1745) and *Botrytis cinerea* (CCF-2361). The results of the in vivo mesocosm experiment with *Mxg* in post-mining metal-contaminated soil revealed that *B. altitudinis* KP-14 strain significantly enhanced plant development and improved biomass production. Overall, the results of the current study suggest that the novel *B. altitudinis* KP-14 strain isolated in this study may be an effective biofertilizer for the cultivation of energy crops under abiotic stress in metal-contaminated soil. However, further in-depth studies are required to evaluate the potential of the *B. altitudinis* KP-14 strain within a mesocosm as well as under field conditions in terms of other physiological parameters, its capacity to improve soil properties, and metals uptake behavior.

## Figures and Tables

**Table 1:** Identification of isolated strains based on their 16S rDNA sequences and NCBI GenBank database.

Isolates	Gram Reaction	Colony Morphology	Nearest Neighbor/Accession Number	% Similarity with Nearest Neighbor	No. of Nucleotides	Assigned NCBI Accession Number
KP-4	Gram-positive	Circular, convex, slimy, white, entire margin	*Bacillus pumilus/*KJ526890	98.4	1463	MN947642
KP-5	Gram-positive	Circular, elevation raised, dry, white Irregular margin	*Bacillus pumilus/*KC692175	98.4	1472	MN947643
KP-9	Gram-positive	Circular, convex, glossy, transparent, entire margin	*Bacillus stratosphericus/*KJ572539	98.5	1466	MN947644
KP-13	Gram-negative	Circular, elevation raised, yellow tint, entire margin	*Stenotrophomonas maltophilia/*DQ113454	98.0	1440	MN947645
KP-14	Gram-positive	Circular, convex, slimy, white, entire margin	*Bacillus altitudinis/*MF511821	100.0	1550	MN966966
KP-16	Gram-negative	Circular, convex, white, entire margin	*Pseudomonas fluorescens/*DQ178227	99.9	996	MN966967
KP-17	Gram-negative	Circular, convex, yellowish white, entire margin	*Pseudomonas fluorescens/*KT695813	98.4	1376	MN947646
KP-18	Gram-positive	Circular, convex, glossy, light yellow, entire margin	*Bacillus* sp./MK571667	99.5	1035	MN947647
KP-19	Gram-negative	Circular, convex, light yellow, entire margin	*Achromobacter* sp./MN515103	99.1	1090	MN947648

**Table 2:** Carbon utilization profile of different bacterial isolates determined by Hi-Carbo Kit (HiMedia, Mumbai, India). + indicates positive (observed) use; − indicates negative use.

Carbon Source	Bacterial Isolate								
	KP-4	KP-5	KP-9	KP-13	KP-14	KP-16	KP-17	KP-18	KP-19
Lactose	+	−	−	−	+	−	−	−	−
Xylose	−	−	−	+	+	+	+	−	−
Maltose	−	−	−	−	+	−	−	−	−
Fructose	+	+	+	−	−	−	−	−	−
Dextrose	+	+	+	+	+	+	+	+	−
Galactose	−	−	+	+	+	−	+	−	−
Raffinose	−	−	−	−	−	−	−	−	−
Trehalose	+	+	+	−	+	−	−	−	−
Melibiose	−	−	−	−	+	+	+	+	−
Sucrose	+	+	+	−	−	+	−	−	−
L-Arabinose	+	+	+	−	+	+	+	−	+
Mannose	+	+	+	+	+	+	+	+	+
Inulin	+	−	+	−	−	−	−	−	−
Sodium gluconate	−	+	−	−	−	−	−	−	−
Glycerol	−	−	−	−	−	−	−	−	−
Salicin	+	+	+	−	−	−	−	−	+
Dulcitol	−	−	−	−	−	−	−	−	−
Inositol	−	−	−	−	−	−	−	−	−
Sorbitol	−	−	−	−	−	−	−	−	−
Mannitol	+	+	+	−	−	−	−	−	−
Adonitol	−	−	−	−	−	−	−	−	−
Arabitol	−	−	−	−	−	−	−	−	−
Erythritol	−	−	−	−	−	−	−	−	−
α-Methyl-D-glucoside	−	−	−	−	−	−	−	−	−
Rhamnose	−	−	−	−	+	+	+	−	−
Cellobiose	+	+	+	−	+	−	−	−	−
Melezitose	−	−	−	−	−	−	−	−	−
α-Methyl-D-mannoside	−	−	−	+	−	−	−	−	−
Xylitol	−	−	−	−	−	−	−	−	−
ONPG	−	−	−	−	−	−	−	−	−
Esculin hydrolysis	+	+	+	+	+	+	−	+	+
D-Arabinose	−	−	−	−	+	−	+	−	−
Citrate	+	+	+	+	+	+	+	+	+
Malonate	+	+	+	+	+	+	+	+	+
Sorbose	−	−	−	−	−	−	−	−	−

**Table 3:** Abiotic stress tolerance including to pH, temperature, salinity, and Pb toxicity exhibited by different isolates.

Abiotic Stresses	Isolated Strains								
	KP-4	KP-5	KP-9	KP-13	KP-14	KP-16	KP-17	KP-18	KP-19
Temperature (°C)									
**4**	−	−	−	+++	+++	++	+++	+++	−
**10**	−	−	−	+++	+++	++	+++	+++	−
**16**	−	−	−	+++	+++	+++	+++	+++	−
**22**	+++	+++	+++	+++	+++	+++	+++	+++	+++
**28**	+++	+++	+++	+++	+++	+++	+++	+++	+++
**37**	+++	+++	+++	−	+++	−	−	+++	+++
**45**	+++	+++	+++	−	+++	−	−	++	++
**50**	++	−	−	−	++	−	−	++	++
**Salinity (%)**									
**0**	+++	+++	+++	+++	+++	+++	+++	+++	+++
**2**	+++	+++	+++	+++	+++	+++	+++	+++	+++
**4**	+++	+++	+++	+++	+++	++	++	+++	+++
**6**	++	+++	+++	−	+++	−	−	+++	+++
**8**	++	+++	+++	−	+++	−	−	+++	+++
**10**	−	++	++	−	+++	−	−	+++	+++
**12**	−	−	−	−	+++	−	−	++	++
**15**	−	−	−	−	++	−	−	−	++
**pH**									
**4**	+++	+++	+++	−	−	−	−	+++	−
**5**	+++	+++	+++	+++	+++	+++	+++	+++	+++
**6**	+++	+++	+++	+++	+++	+++	+++	+++	+++
**7**	+++	+++	+++	+++	+++	+++	+++	+++	+++
**8**	+++	+++	+++	+++	+++	+++	+++	+++	+++
**Pb toxicity (ppm)**									
**100**	+++	+++	+++	+++	+++	+++	+++	+++	+++
**300**	+++	+++	+++	+++	+++	+++	+++	+++	+++
**500**	+++	+++	+++	+++	+++	+++	+++	+++	+++
**700**	+++	+++	+++	+++	+++	+++	+++	+++	+++
**1000**	−	−	−	−	+++	−	−	+++	+++
**1200**	−	−	−	−	+	−	−	+	+
**1500**	−	−	−	−	−	−	−	−	−

Note: negative (−) for no growth, moderately positive (++) for more growth but less than the control plates, and strongly positive (+++) for growth similar to the control plates.

**Table 4:** Plant growth-promoting characteristics exhibited by selected isolates: KP-14, KP-18, and KP19.

Isolate	P Solubilization (µg/mL)	Indole Acetic Acid (IAA) (µg/mL)	1-Aminocyclopropane-1-Carboxylate) (ACC) Deaminase Activity	Ammonia	Siderophore	Hydrogen Cyanide (HCN)	Antifungal Activity	
							*Fusarium culmorum* (CCF-1745)	*Botrytis cinerea* (CCF-2361)
KP-14	130.0 ± 3.2	27.8 ± 0.8	+	+	+	+	+	+
KP-18	14.5 ± 2.9	10.5 ± 0.2	−	+	+	+	+	−
KP-19	32.8 ± 1.2	1.1 ± 0.7	−	+	+	−	−	−

Note: −, negative; +, positive.

**Table 5:** PGP attributes and the abiotic stress tolerance exhibited by different *Bacillus* sp.

Organism	Salt Tolerance (%)	Thermo Tolerance (°C)	P Solubilization	IAA	ACC	Ammonia	Siderophore	HCN	Reference
*B. subtilis* AURB65	5	60	+	*	*	*	*	+	[81]
*B. altitudinis* BRHS/S-73	*	*	+	+	*	*	+	+	[63]
*Bacillus* sp. EL1	2	*	−	+	−	*	*	*	[68]
*Bacillus* sp. NIASMIII	12	*	−	+	−	*	+	*	[82]
*B. licheniformis* HSW-16	11	*	+	+	+	*	−	*	[67]
*Bacillus* sp. SR-2-1/1	10	*	+	+	+	*	*	*	[74]
*B. subtilis* GSW-E-6	2	*	−	+	+	*	+	*	[29]
*B. tequilensis* SSB07	*	35	*	+	*	*	*	*	[83]
*B. subtilis* RH5	10	45	+	+	*	+	+	+	[26]
*B. pumilus* HL3RS14	20	*	+	+	*	*	−	−	[84]
*B. cereus* TCR17	*	50	+	+	*	*	+	*	[41]
*B. proteolyticus* 4D	*	*	+	+	−	+	−	*	[65]
*B. velezensis* 9I	*	*	+	+	−	+	+	*	[65]
*B. altitudinis* KP-14	15	50	+	+	+	+	+	+	This study

Note: *, not mentioned; +, positive; −, negative.

## Supplementary Materials

The following are available online at https://www.mdpi.com/2079-7737/9/9/305/s1, Table S1: Agrochemical characteristics of soil collected from the post-mining metal-contaminated site, Table S2: Elemental analysis of soil collected from the post-mining metal-contaminated site, Table S3: Statistical analysis of *B. altitudinis* KP-14 effect on germination index of *B. alba* seeds, Table S4: Statistical analysis of *B. altitudinis* KP-14 effect on root and shoot length of *B. alba*, Table S5: Statistical analysis of *B. altitudinis* KP-14 effect on growth of *Mxg* i.e.,: (a) Two way ANOVA for height and vegetation time, (b) One way ANOVA for leaves dry mass, (c) One way ANOVA for stems dry mass, (d) One way ANOVA for roots dry mass.
