# Supplementary material for: Bioprospecting of a Novel Plant Growth-Promoting Bacterium Bacillus altitudinis KP-14 for Enhancing Miscanthus × giganteus Growth in Metals Contaminated Soil
Source: Biology (Basel). 2020 Sep 22;9(9):305. doi: 10.3390/biology9090305 (PMC7564662; doi:10.3390/biology9090305)
Supplement: Supplementary file 1 [file biology-09-00305-s001.pdf]

Article title: Bioprospecting of a novel plant growth-promoting bacterium *Bacillus altitudinis* KP-14 for enhancing *Miscanthus* × *giganteus* growth in metal-contaminated soil
Journal name: Biology
Author names: Kumar Pranaw*, Valentina Pidlisnyuk, Josef Trögl and Hana Malinská

Table S1. Agrochemical characteristics of soil collected from the post-mining metals contaminated site.

**Macro elements**	N-Nitrogen of NO_3 (mg/Kg)	21.4
	N-Nitrogen of Alkali hydrolysed (mg/Kg)	108.0
	P- Phosphorus (A/c to Kirsanov) (mg/Kg)	50.0
	K- Potassium (A/c to Kirsanov) (mg/Kg)	600.0
**General indicators**	Organic matter (%)	3.4
	pH of water extraction	5.0
	pH of salt extraction	3.7
	General salt content (mg/100g)	48.0
	Electrical conductivity (mS/cm)	0.1
	Hydrolytical acidity (mmol-equivalent/100g)	17.3
	Sum of exchange alkali (mmol-equivalent/100g)	10.0
**Micro elements**	Sulphur (mg/Kg)	1.5
	Calcium exchangeable (mmol-equivalent/100g)	16.0
	Magnesium exchangeable (mmol-equivalent/100g)	2.3

Table S2. Elemental analysis of soil collected from post-mining metals contaminated site.

Elements	Concentration (in mg/kg)
Magnesium (Mg)	11999.5
Aluminium (Al)	120212.4
Silica (Si)	286948.8
Phosphorus (P)	647.4
Sulphur (S)	949.6
Potassium (K)	17506.1
Cancium (Ca)	7745.1
Titanium (Ti)	16224.4
Vanadium (V)	372.6
Chromium (Cr)	201.7
Manganese (Mn)	523.4
Iron (Fe)	51862.8
Nickel (Ni)	73.0
Copper (Cu)	50.3
Zinc (Zn)	147.4
Galium (Ga)	34.2
Selenium (Se)	-
Rubidium (Rb)	124.0
Strontium (Sr)	305.4
Y	31.5
Zr	539.7
Nb	115.3
Pb	53.7

Table S3. Statistical analysis of *B. altitudinis* KP-14 effect on % germination of *B. alba* seeds.

ANOVA: Single Factor

SUMMARY

Groups	*Count*	*Sum*	*Average*	*Variance*
Uninoculated control	5	340	68	120
Inoculated treatment	5	450	90	100

ANOVA

Source of Variation	*SS*	*df*	*MS*	*F*	*P-value*	*F crit*
Between Groups	1210	1	1210	11	0.0105932	5.31766
Within Groups	880	8	110			
Total	2090	9				

Table S4. Statistical analysis of the *B. altitudinis* KP-14 effect on root and shoot length of *B. alba*.

ANOVA: Two-Factor with Replication

SUMMARY	Root Length	Shoot Length	Total			
Uninoculated control						
Count	5	5	10			
Sum	6.9	5.4	12.3			
Average	1.38	1.08	1.23			
Variance	0.182	0.032	0.12011			
Inoculated treatment						
Count	5	5	10			
Sum	22.9	12	34.9			
Average	4.58	2.4	3.49			
Variance	1.712	0.3	2.21433			
Total						
Count	10	10				
Sum	29.8	17.4				
Average	2.98	1.74				
Variance	3.68622222	0.63155556				
ANOVA						
Source of Variation	*SS*	*df*	*MS*	*F*	*P-value*	*F crit*
Sample	25.538	1	25.538	45.8904	0.0000045	4.494
Columns	7.688	1	7.688	13.8149	0.0018745	4.494
Interaction	4.418	1	4.418	7.9389	0.0123831	4.494
Within	8.904	16	0.5565			
Total	46.548	19				

Table S5. Statistical analysis of the *B. altitudinis* KP-14 effect on growth i.e., (a) Two way ANOVA for height and vegetation time, (b) One way ANOVA for leaves dry mass, (c) One way ANOVA for stem dry mass, (d) One way ANOVA for roots dry mass, of *Mxg*.

**a) ANOVA: TWO-FACTOR (Height and Vegetation period) WITH REPLICATION**						
SUMMARY	2 month	3 month	4 month	5 month	6 month	Total
C						
Count	4	4	4	4	4	20
Sum	486	486	520	534	534	2560
Average	121.5	121.5	130	133.5	133.5	128
Variance	8.33333	8.33333	48	120.333	120.3333333	79.5789
M						
Count	4	4	4	4	4	20
Sum	517	540	614	630	630	2931
Average	129.25	135	153.5	157.5	157.5	146.55
Variance	20.9167	29.3333	89	99	99	204.997
Total						
Count	8	8	8	8	8	
Sum	1003	1026	1134	1164	1164	
Average	125.375	128.25	141.75	145.5	145.5	
Variance	29.6964	68.2143	216.5	258.571	258.5714286	
ANOVA						
Source of Variation	*SS*	*df*	*MS*	*F*	*P-value*	*F crit*
Sample	3441.03	1	3441.03	53.5499	0.00000004	4.17088
Columns	3027.1	4	756.775	11.7771	0.00000720	2.68963
Interaction	452.1	4	113.025	1.75892	0.16327767	2.68963
Within	1927.75	30	64.2583			
Total	8847.98	39				

(b) ANOVA: Single Factor i.e., Leaves dry mass after harvesting

SUMMARY						
Groups	*Count*	*Sum*	*Average*	*Variance*		
C	4	43.98	10.995	3.22403		
M	4	65.47	16.3675	1.35209		
ANOVA						
Source of Variation	*SS*	*df*	*MS*	*F*	*P-value*	*F crit*
Between Groups	57.7275	1	57.7275	25.2299	0.0024	5.98738
Within Groups	13.7284	6	2.28806			
Total	71.4559	7				

(c) ANOVA: Single Factor i.e., Stem dry mass after harvesting

SUMMARY

Groups	*Count*	*Sum*	*Average*	*Variance*
C	4	36.82	9.205	10.0467
M	4	68.42	17.105	4.2319

ANOVA

Source of Variation	*SS*	*df*	*MS*	*F*	*P-value*	*F crit*
Between Groups	124.82	1	124.82	17.4835	0.0058	5.98738
Within Groups	42.8358	6	7.1393			
Total	167.656	7				

(d) ANOVA: Single Factor i.e., Roots dry mass after harvesting

SUMMARY

Groups	*Count*	*Sum*	*Average*	*Variance*
C	4	108.54	27.135	39.8216
M	4	190.6	47.65	4.22453

ANOVA

Source of Variation	*SS*	*df*	*MS*	*F*	*P-value*	*F crit*
Between Groups	841.73	1	841.73	38.2204	0.00082	5.98738
Within Groups	132.139	6	22.0231			
Total	973.869	7				
